# Incidence and outcomes of extubation failure in mechanically ventilated patients with cirrhosis: a post-hoc analysis of a prospective multicenter study

**DOI:** 10.1186/s13613-025-01576-3

**Published:** 2025-10-16

**Authors:** Yassir Aarab, Joris Pensier, Fanny Garnier, Clement Monet, Ines Lakbar, Gerald Chanques, Audrey de Jong, Mathieu Capdevila, Samir Jaber

**Affiliations:** 1 Anesthesiology and Intensive Care Unit, Anesthesia and Critical Care Department B, INSERM U1046, CNRS UMR 9214, Saint Eloi Teaching Hospital, PhyMedExp, University of Montpellier, 80 Avenue Augustin Fliche, 34295 Montpellier, Cedex 5, France; 2 Intensive Care Unit, Clinique Saint-Jean Sud de France, Montpellier, France

**Keywords:** Cirrhosis, Extubation failure, Intensive care unit, Mechanical ventilation

## Abstract

**Background:**

Patients with comorbidities who are liberated from invasive mechanical ventilation could be at risk of extubation failure in the intensive care unit (ICU). Incidence and associated outcomes of reintubation in patients with cirrhosis have been poorly studied. We aimed to evaluate the incidence, causes and mortality of reintubation in patients with cirrhosis.

**Methods:**

We conducted a post hoc analysis of the French prospective multicenter observational trial (FREE-REA) evaluating the incidence and risk factors of extubation failure in 26 ICUs. The primary outcome was the incidence of extubation failure defined as the need for reintubation within 7 days after extubation. Secondary outcomes were the incidence of reintubation at 48 h, the causes and risk factors of extubation failure, ICU length of stay and in-hospital mortality. We compared patients with cirrhosis and patients without cirrhosis.

**Results:**

Of the 1,443 analyzed patients, 165 (11%) had cirrhosis. The incidence of reintubation within 7 days was 21% (34/165) in patients with cirrhosis and 13% (167/1278) in patients without cirrhosis (*p* < 0.01). Reintubation at 48 h was not significantly different between patients with cirrhosis and patients without cirrhosis (9% versus 10%, *p* = 0.55). Admission for shock was identified as the only independent risk factor for extubation failure in multivariate analysis [OR 3.24, 95% CI (1.24–8.44), *p* = 0.02]. In patients with extubation failure, ICU length of stay was significantly longer in patients with cirrhosis compared to those without (28 ± 25 versus 18 ± 12 days, *p* < 0.01); In-hospital mortality was higher in extubation failure patients with cirrhosis in comparison to patients without cirrhosis without reaching significance (16/34 (47%) versus 51/167 (31%), *p* = 0.06).

**Conclusion:**

Extubation failure was significantly higher in patients with cirrhosis compared to patients without cirrhosis. A trend for higher in-hospital mortality was observed in reintubatedpatients with cirrhosis. Neurologic failure was the main cause for reintubation at 48 h in patients with cirrhosis.

**Clinical trials:**

The study was registered on clinicaltrials.gov (identifier no. NCT02450669). Registered 01/12/2013.

**Supplementary Information:**

The online version contains supplementary material available at 10.1186/s13613-025-01576-3.

## Background

Patients with cirrhosis frequently experience acute decompensations and develop organ failures leading to intensive care unit (ICU) admission [1]. Invasive mechanical ventilation (IMV) is then required for 69% of these patients [2, 3]. Intubated ICU patients with cirrhosis exhibit high mortality rates ranging from 50 to 90% [4–6]. Thus, limited attention has been specifically directed toward investigating extubation failure among cirrhotic patients admitted to the ICU [7, 8].

Extubation failure is usually defined as the need for reintubation within 48–72 h [9–11]. This time frame is often extended to 7 days, especially when noninvasive ventilation (NIV) or high-flow nasal oxygen (HFNO) are used after extubation [12–14]. Reintubation is associated with extended IMV duration, increased tracheostomy requirement, augmented healthcare expenditures, and increased mortality rates [9, 15]. Specific subgroups of patients are known to have a higher incidence of reintubation (i.e., patients with Chronic Obstructive Pulmonary Disease (COPD), brain injury, obesity, chronic cardiac disease) [12–14, 16–18]. Patients with cirrhosis exhibit specificities (hepatic encephalopathy, ascites, sarcopenia) that may hamper prognosis differently after extubation [1].

To our knowledge, no published data has specifically investigated the issue of extubation failure in patients with cirrhosis within a multicenter cohort of critically ill patients.

We hypothesized that the incidence of extubation failure would be higher in patients with cirrhosis compared to patients without cirrhosis. We also speculated that patients experiencing extubation failure who have cirrhosis would experience worse outcomes in comparison to those without cirrhosis.

In this *post-hoc* analysis of the FREE-REA study, we aimed to compare the incidence, causes, risk factors, and outcomes of extubation failure among a cohort of mechanically ventilated patients with and without cirrhosis [19].

## Methods

### Study design and population

We conducted a *post-hoc* analysis of the FREE-REA French prospective, observational, multicenter study performed in 26 intensive care units (ICU) from December 2013 to May 2015 [19]. Briefly, all consecutive adult patients extubated in participating ICUs were included. Exclusion criteria were age < 18 years, pregnancy, lack of informed consent, and extubation with a do-not-reintubate order. In patients undergoing more than one extubation procedure, only the first episode was considered. Data collection and additional methodological details were previously described [19].

### Ethics and consent

The appropriate IRB (*Comité de Protection des personnes Sud-Mediterranée III*) approved the study protocol (code UF: 9242, register: 2013-A01402-43). The study was registered on clinicaltrials.gov (identifier no. NCT 02450669). We followed the STROBE guidelines for observational studies [20].

### Categorization of patients and definition of extubation failure

Patients were categorized based on known history of cirrhosis into patients “with cirrhosis” and “without cirrhosis”. As performed in previous studies, the diagnosis of cirrhosis was based on previous histology findings when available or on various associations of clinical, biological, endoscopic, and/or ultrasonographic or imaging findings, including cutaneous manifestations such as jaundice or skin telangiectasias, evidence of portal hypertension such as variceal bleeding, ascites, hepatic encephalopathy, and biological results of liver failure [5, 6]. For each patient, “extubation failure” was defined as the need for reintubation within 7 days after extubation. Extubation success was defined as no need for reintubation within 7 days after extubation.

### Outcomes

The primary outcome was the incidence of extubation failure defined as the need for reintubation within 7 days.

The secondary outcomes were: reintubation at 48 h, the length of IMV during the ICU stay, the need for curative NIV, vasopressors or renal replacement therapy, the occurrence of hospital-acquired infections (pneumonia, catheter infection, bacteremia, urinary infection), the characteristics of the extubation procedures, the ICU length of stay, the in-hospital length of stay and the in-hospital mortality.

We compared patients with and without cirrhosis for all outcomes. Then, we compared patients with and without extubation failure.

### Statistical analysis

Quantitative variables were expressed as means (standard deviation) or medians (interquartile range [25th-75th percentile]) based on normality, assessed using the Shapiro–Wilk test. Comparisons between groups were performed using the Student's t-test or Mann–Whitney U test as appropriate. Categorical variables were presented as numbers (percentages) and compared using the chi-square test or Fisher's exact test, as appropriate. Missing data were imputed using multiple imputation by chained equations, with the number of imputations determined based on the proportion of missing data. Analysis were performed in patients with and without cirrhosis.

First, descriptive statistics were calculated for the overall cohort, and for patients with and without cirrhosis. The incidence of reintubation within 7 days after extubation (extubation failure) was calculated among patients with and without cirrhosis, then compared using the chi-square test.

Second, univariate analyses were performed to compare outcomes between patients with and without cirrhosis, and to identify potential risk factors for reintubation at day 7. Variables with a *p*-value < 0.20 in the univariate analysis were considered for inclusion in the multivariable model.

Third, a multivariate logistic regression model was established to identify independent risk factors for reintubation within 7 days after extubation. All variables with *P*-values less than 0.20 in the univariate logistic regression analysis were entered into the multivariate logistic model and a backward stepwise selection procedure was used to select the final model according to the Bayesian information criterion. Interactions between variables were tested.

Fourth, survival curves for mortality within 60 days were generated using the Kaplan–Meier method and compared using the log-rank test. Two sensitivity analysis were performed in patients with cirrhosis and without cirrhosis, based on duration of IMV (≤ 24 h vs. > 24 h) and based on the reason for ICU admission (post-operative vs. medical).

Finally, the characteristics of the extubation procedures were compared in patients with and without cirrhosis.

All statistical analyses were performed using R software (version 4.2.0). A *p*-value < 0.05 was considered statistically significant.

## Results

### Description

**Fig. 1:**
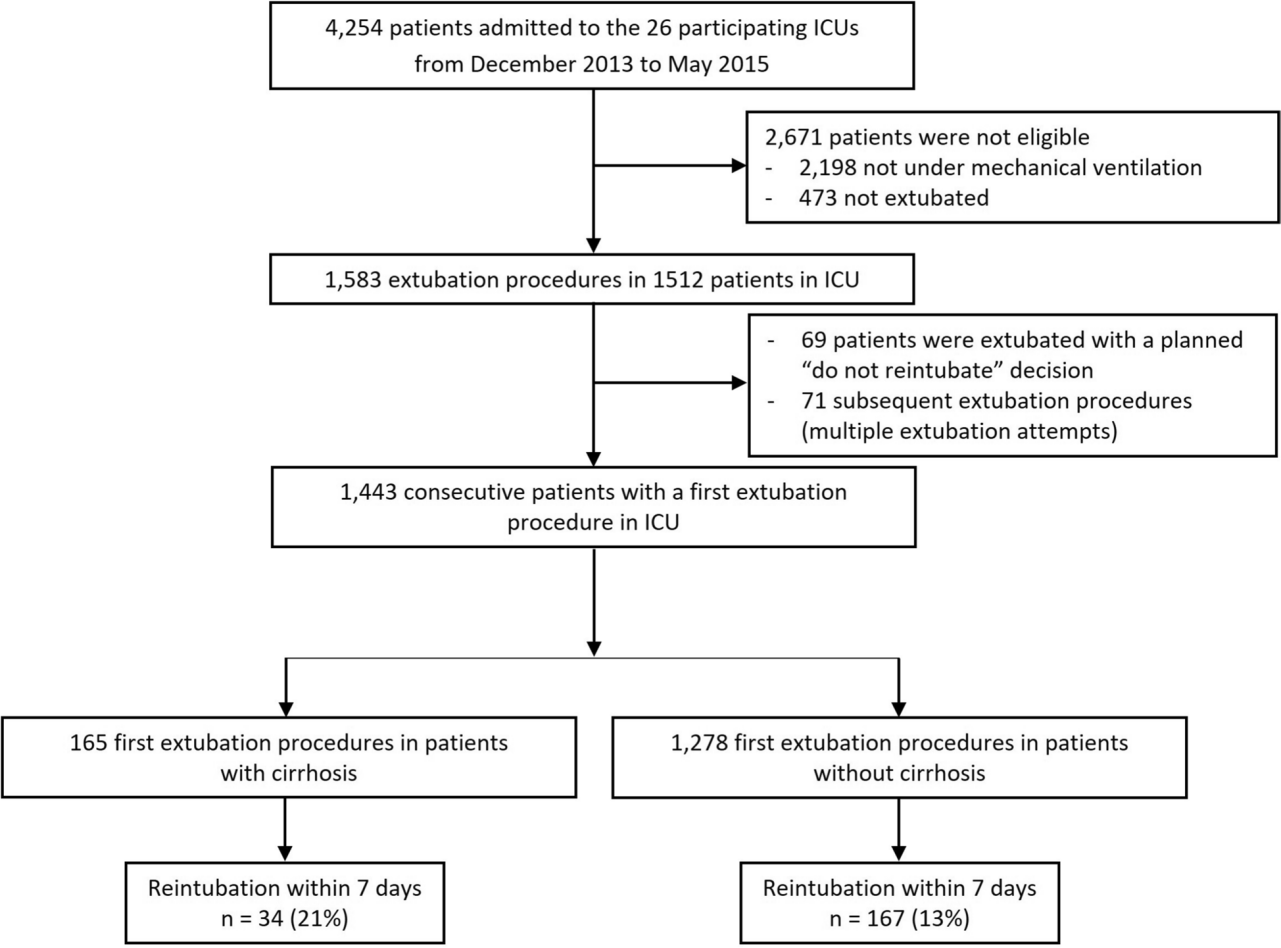
Flow chart of the study. This figure shows the flow chart of patients admitted to participating units during the study. Consecutive patients with a first extubation procedure were included. *ICU*: Intensive Care Unit

Over a 18-month period, 1,443 patients were included in the analysis including 165 patients with cirrhosis (11%) (Fig. 1). Baseline characteristics, primary reasons for admission and intubation for patients with cirrhosis are presented in Table 1. Overall, we recorded 201/1,443 (14%) episodes of reintubation within 7 days after extubation. Table S1 reported baseline characteristics according to cirrhosis status.

**Table 1:** Baseline characteristics in patients with cirrhosis compared between those who required reintubation and those who were successfully extubated

Characteristic	*Overall* *(n* = *165)*			
		Extubation success (n = 131)	Extubation failure (n = 34)	*p*-value
Age, mean (SD)	58 (10)	57 (10)	59 (12)	0.41
Female sex, n (%)	43 (26%)	33 (25%)	10 (29%)	0.78
SAPS2, mean (SD)	45 (15)	45 (15)	48 (13)	0.34
SOFA score before extubation, mean (SD)	6 (6)	6 (6)	6 (5)	0.83
BMI (kg/m^2^), mean (SD)	26.1 (4.8)	26.1 (4.7)	26.0 (5.1)	0.94
BMI ≥ 30 kg/m^2^, n (%)	28 (17%)	22 (17%)	6 (18%)	1.00
*Type of admission*				0.79
Medical, n (%)	87 (53%)	68 (52%)	19 (56%)	
Surgical, n (%)	78 (47%)	63 (48%)	15 (44%)	
Smoking, n (%)	59 (36%)	47 (36%)	12 (35%)	1.00
Alcohol abuse, n (%)	101 (61%)	80 (61%)	21 (62%)	1.00
COPD, n (%)	26 (16%)	23 (18%)	3 (8.8%)	0.32
Chronic renal disease, n (%)	38 (23%)	27 (21%)	11 (32%)	0.22
Chronic heart disease, n (%)	13 (7.9%)	12 (9.2%)	1 (2.9%)	0.31
*Primary reason for ICU admission, n (%)*				
Acute respiratory failure	25 (15%)	24 (18%)	1 (2.9%)	0.05
Post-operative	71 (43%)	59 (45%)	12 (35%)	0.41
Neurologic failure	20 (12%)	17 (13%)	3 (8.8%)	0.71
Hemodynamic instability	34 (21%)	21 (16%)	13 (38%)	< 0.01
Others	15 (9%)	10 (8%)	5 (15%)	0.21
*Primary reason for intubation, n (%)*				
Acute respiratory failure	35 (21%)	26 (20%)	9 (26%)	0.54
Neurologic failure	25 (15%)	19 (15%)	6 (18%)	0.85
Hemodynamic instability	16 (10%)	10 (8%)	6 (18%)	0.15
Cardiac arrest	2 (1%)	2 (1%)	0 (0%)	1.00
Surgery	57 (35%)	47 (36%)	10 (29%)	0.48
Others	30 (18%)	27 (20%)	3 (9%)	0.11
Length of intubation before extubation (days), median (IQR)	2 [1–5]	2 [1–4]	2 [1–6]	0.26

### Primary outcome

The incidence of extubation failure defined as reintubation within 7 days after extubation was significantly higher in patients with cirrhosis compared to those without cirrhosis: 34/165 (21%) versus 167/1278 (13%) [OR 1.73, 95% CI (1.15–2.60), *p* < 0.01].

### Secondary outcome

The incidence of reintubation at 48 h did not significantly differ between patients with and without cirrhosis: 14/165 (9%) versus 132/1278 (10%) [OR 0.80, 95% CI (0.45–1.42), *p* = 0.55].

Sensitivity analyses were performed on patients ventilated for more or less than 24 h and on patients admitted for post-operative or medical reason (Table 2).

**Table 2:** Incidence of extubation failure within 7 days compared between patients with and without cirrhosis—sensitivity analysis of primary outcome

Outcome	Population	Patients with cirrhosis	Patients without cirrhosis	*p*-value
Overall population, n	1443	165	1278	
Extubation failure, n (%)	201 (14%)	34 (21%)	167 (13%)	0.01
Sensitivity analysis with length of IMV ≤ 24 h, n	523	67	456	
Extubation failure, n (%)	59 (11%)	16 (24%)	43 (9%)	< 0.01
Sensitivity analysis with length of IMV > 24 h, n	920	98	822	
Extubation failure, n (%)	142 (15%)	18 (18%)	124 (15%)	0.39
Sensitivity analysis in medical patients, n	946	94	852	
Extubation failure, n (%)	151 (16%)	22 (23%)	129 (15%)	0.04
Sensitivity analysis in post-operative patients	497	71	426	
Extubation failure, n (%)	50 (10%)	12 (17%)	38 (9%)	0.04

IMV Invasive Mechanical Ventilation

**Fig. 2:**
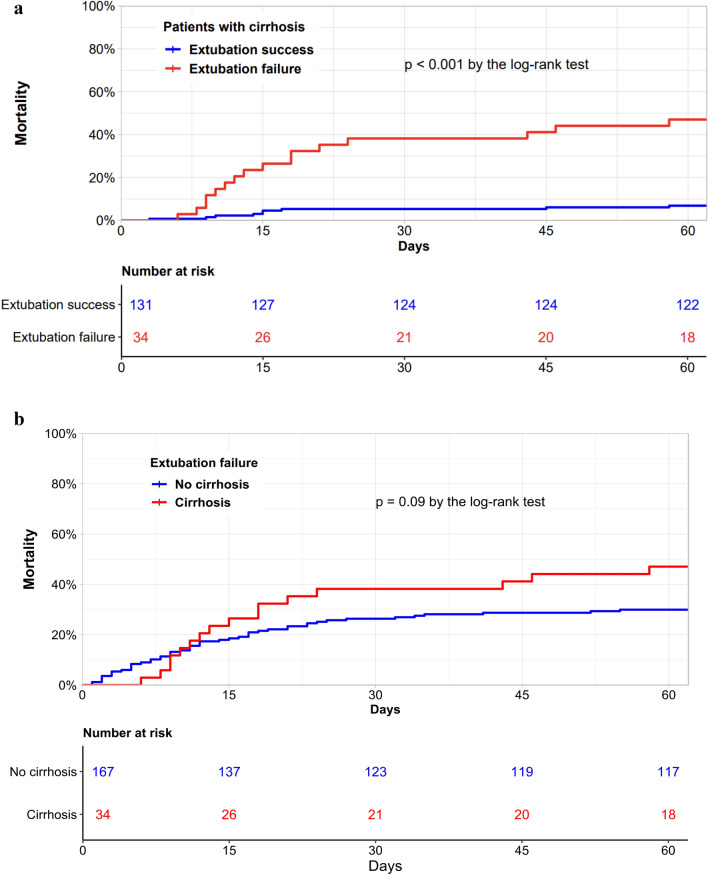
Cumulative incidence of mortality within the 60 days following extubation. **A** Cumulative incidence of mortality within the 60 days following extubation among patients with cirrhosis compared between those who required reintubation (red) and those who were successfully extubated (blue) (n = 165). **B** Cumulative incidence of mortality within the 60 days following extubation compared between patients with cirrhosis (red) and those without cirrhosis (blue) who required reintubation (n = 201)

First, we compared patients with cirrhosis according to extubation success or failure (Table 3). Unsurprisingly, reintubated patients showed worse outcomes, notably significantly higher mortality (47% versus 7%, *p* < 0.01). Figure 2A reported the cumulative incidence of mortality within the 60 days following extubation among patients with cirrhosis, comparing those who required reintubation and those who were successfully extubated (log rank test, *p* < 0.01). Sensitivity analyses found similar results (Figure S1).

**Table 3:** Secondary outcomes according to extubation failure or success among patients with cirrhosis

Outcomes	Overall (n = 165)	Extubation success (n = 131)	Extubation failure (n = 34)	*p*-value
Total length of IMV (days), median (IQR)	3 [1–8]	3 [1–6]	10 [4–16]	< 0.01
Hospital-acquired infections, n (%)	45 (27%)	23 (18%)	22 (65%)	< 0.01
Pneumonia	16 (10%)	4 (3%)	12 (35%)	< 0.01
Catheter	4 (2%)	4 (3%)	0 (0%)	0.69
Bloodstream	17 (10%)	11 (8%)	6 (18%)	0.21
Urinary tract	8 (5%)	4 (3%)	4 (12%)	0.10
Use of vasopressor, n (%)	29 (17%)	11 (8%)	17 (50%)	< 0.01
Use of renal replacement therapy, n (%)	12 (7%)	6 (5%)	6 (18%)	0.03
Use of curative NIV after extubation, n (%)	49 (30%)	33 (25%)	16 (47%)	0.02
ICU length of stay (days), median (IQR)	10 [5–16]	8 [5–13]	17 [12–36]	< 0.01
In-hospital length of stay (days), median (IQR)	25 [17–46]	23 [16–40]	34 [18–58]	< 0.01
In hospital mortality, n (%)	25 (15%)	9 (7%)	16 (47%)	< 0.01

ICU: Intensive Care Unit, IMV Invasive Mechanical Ventilation, IQR Interquartile range, NIV Non Invasive Ventilation,

“Total length of IMV” refers to the cumulative duration of invasive mechanical ventilation during the entire ICU stay. “Use of vasopressor” and “Use of renal replacement therapy” refers to the initiation or continuation of these supports exclusively after extubation and until ICU discharge. “Hospital-acquired infections” were recorded if they occurred after extubation

Among patients who experienced reintubation within 7 days (extubation failure), we compared patients with and without cirrhosis (Table S2). Patients with cirrhosis had a significantly higher use of vasopressors after extubation (50% versus 25%, *p* < 0.01), a significantly longer ICU length of stay (*p* < 0.01) and a significantly longer in-hospital length of stay (*p* < 0.01). In-hospital mortality was also higher among patients with cirrhosis but did not reach statistical significance (47% versus 31%, *p* = 0.06)**. **Figure 2B reported the cumulative incidence of mortality within 60 days following extubation, comparing patients with cirrhosis and those without cirrhosis who required reintubation (log rank test, *p* = 0.09). Table S3 compared outcomes in patients with and without cirrhosis.

### Causes and risk factors for extubation failure

The only specific independent risk factor for extubation failure in patients with cirrhosis in the final multivariate model was ICU admission for shock [OR 3.24 (1.24–8.44), *p* = 0.02] **(**Table 4**)**. The main cause of reintubation at 48 h in patients with cirrhosis was neurologic failure: 5/14 (36%) (Table S4).

**Table 4:** Risk factors for extubation failure. Multivariate analysis

Characteristic	Odd ratio	95% CI	*p*-value
*Patients with cirrhosis*			
SOFA ≥ 8	1.93	[0.80–4.64]	0.24
Admission for acute respiratory failure	0.19	[0.01–1.04]	0.06
Admission for shock	3.24	[1.24–8.40]	0.02
*Patients without cirrhosis*			
Female sex	0.76	[0.52–1.12]	0.17
SOFA ≥ 8	2.75	[1.38–5.25]	< 0.01
Admission for acute respiratory failure	1.77	[1.15–2.7]	0.01
Admission for neurologic failure	1.57	[1.01–2.42]	< 0.05
Length of intubation ≥ 8 days	1.35	[0.86–2.07]	0.19
Moderate to strong cough strength	0.57	[0.38–0.87]	< 0.01

CI: Confidence Interval, SOFA: Sequential Organ Failure Assessment,

Variables included in the multivariable model are these with *p* < 0.20

In patients with cirrhosis, acute respiratory failure, shock, and pressure support during weaning test were entered into the multivariable analysis

In patients without cirrhosis, sex, SOFA score, postoperative vs medical admission, chronic heart disease, acute respiratory failure, neurologic failure, length of ventilation, pressure support during weaning test, and preventive NIV after extubation were entered into the multivariable analysis

### Extubation procedure

The characteristics of extubation procedures and spontaneous breathing trial (SBT) according to cirrhosis status and extubation failure or success are presented in Table S5. A SBT was performed in 124/165 (75%) and 1021/1278 (80%) patients with cirrhosis and without cirrhosis respectively (*p* = 0.22). In patients with cirrhosis, a SBT with pressure support was more frequently used (85/165 (51%) versus 525/1278 (41%), *p* < 0.01). Precautions for reintubation were significantly less frequent in patients with cirrhosis compared to those without cirrhosis: reintubation kit ready (28/165 (17%) versus 508/1278 (40%), *p* < 0.01) and the use of prophylactic NIV (23/165 (14%) versus 306/1278, (24%), *p* < 0.01). When comparing patients with cirrhosis according to extubation failure or success, we found no significant difference in these precautions. In contrary, more precautions were implemented for patients without cirrhosis who finally experienced reintubation: physiotherapy (*p* < 0.01) and prophylactic NIV after extubation (*p* < 0.01).

## Discussion

The main result of this *post-hoc* analysis of a prospective observational multicenter cohort is that patients with cirrhosis exhibit a significantly higher incidence of reintubation within 7 days after extubation compared to patients without cirrhosis. In patients with cirrhosis, the unique specific risk factor for extubation failure was ICU admission for shock. ICU length of stay and in-hospital stay were significantly longer in extubation failure patients with cirrhosis compared to those without cirrhosis. There was also a trend for higher in-hospital mortality in reintubated patients with cirrhosis. The main cause for reintubation at 48 h was neurologic failure.

Patients with cirrhosis represent a distinct population among IMV patients to wean [1]. Indeed, extubation failure in patients with cirrhosis could be explained by potentially modifiable factors (ascites, hydrothorax, encephalopathy, pulmonary infections, volume overload) and by non-modifiable factors (sarcopenia, nutritional deficiency, hepato-pulmonary syndrom, and porto-pulmonary hypertension) [1, 6, 21–26]. However, the field of weaning from IMV and extubation in cirrhotic patients remains inadequately investigated. To date, only 3 studies have indirectly investigated extubation failure within 48–72 h in critically ill patients with cirrhosis [7, 8, 27]. Sasso et al. evaluated the mortality among 113 patients with cirrhosis under IMV (53%), and reported as a secondary outcome a 29% extubation failure rate [7]. Gibbs et al. documented retrospectively an 18% rate of extubation failure in selected patients with cirrhosis and hepatic coma [7]. Sahu et al. evaluated a weaning index in 27 patients with cirrhosis who reached SBT and reported a 33% extubation failure rate [27]. Our reported rates of extubation failure in patients with cirrhosis from a prospective multicenter cohort were lower when considering reintubation within 48 h (9%) but quite similar when considering reintubation within 7 days (21%), underlining the critical importance of a consistent definition of extubation failure.

Our analysis reveals a divergence in reintubation patterns. This suggests a disproportionate increase in reintubations occurring between days 3 and 7 post-extubation in patients with cirrhosis. This finding warrants further consideration as it points to potential delayed complications impacting respiratory function. Specifically, the development or worsening of hepatic encephalopathy, ascites, or an increased susceptibility to infection in the days following extubation could contribute to airway or non airway extubation failure and need for reintubation in this vulnerable population. The presence of sarcopenia and progressive respiratory muscle fatigue, common in cirrhosis, may also explain this delayed need for reintubation. Future studies should focus on detailed daily monitoring of respiratory parameters and cirrhosis-related complications post-extubation to better understand the underlying mechanisms driving this increased risk during the 3–7 day window. Furthermore, we report herein for the first time that the primary cause of extubation failure at 48 h in selected patients with cirrhosis was neurologic failure (36%), followed by non-cardiac respiratory failure (21%), contrasting with the hemodynamic instability more often seen in patients without cirrhosis. While limited by sample size and database constraints, exploratory analyses comparing outcomes based on the timing of reintubation (within 48 h vs. days 3–7) are presented in Tables S6 and S7.

Interestingly, while patients with cirrhosis experienced a higher reintubation rate, we did not observe a statistically significant difference in overall mortality compared to patients without cirrhosis (Table S3). Our study population represents a carefully selected subset of patients deemed stable enough for an extubation attempt, who likely represent a population with a more favorable overall prognosis than the broader population of critically ill patients with cirrhosis [2, 5]. This pre-selection likely attenuated the impact of reintubation on overall mortality. However, it is important to note that we observed a trend toward a significant mortality difference between reintubated patients with and without cirrhosis (Table S2). Moreover, sensitivity analysis based on duration of IMV (≤ 24 h vs. > 24 h) and based on the reason for ICU admission (post-operative vs. medical) found similar results. Larger studies focusing on this specific population are warranted to confirm this trend.

Compared to other high-risk populations, our cohort of patients with cirrhosis showed similar rates of extubation failure [12–14, 28]. Recent findings from randomized trials including high-risk patients indicated extubation failure rates ranging from 9 to 15% within 48 h and between 21 to 31% within seven days [12–14, 28]. These disparities have been partly explained by the varied duration of IMV before extubation and by the use of NIV, HFNO, or neither after extubation. In our study, duration of IMV before extubation did not differ in patients with cirrhosis (Table 1). The curative use of NIV was not different between patients with and without cirrhosis who experienced extubation failure (Table S2). When comparing extubation failure or success in patients with cirrhosis, the curative use of NIV was higher in reintubated patients (47% versus 25%, *p* < 0.01) (Table 3).

Interestingly, disparities emerged in the pre and post-extubation management between patients with and without cirrhosis. In patients with cirrhosis, before extubation, the assessment of readiness for weaning was more frequently conducted using an easiest SBT with pressure support (51% versus 41%, *p* < 0.01), and the preparation of a reintubation kit was less common compared to patients without (*p* < 0.01) cirrhosis (Table S5). After extubation, the immediate prophylactic use of NIV was significantly less prevalent in patients with cirrhosis (14% versus 24%, *p* < 0.01). Furthermore, when comparing extubation success and failure in patients with cirrhosis specifically, no differences were noted for these precautions in patients with cirrhosis. Conversely, non-cirrhotic patients showed a greater implementation of precautions for those who finally failed extubation (Table S5). These findings suggests potential differences in perceived risk and management approaches for extubation in patients with cirrhosis. However, these observations are based on a non-randomized, observational study, and do not establish causal relationships.

Extubation failure has consistently been associated with increased mortality [9, 15, 19]. In the original FREE-REA study, in-hospital mortality rates were 29% and 10%, respectively, for unselected patients with extubation failure within 48 h and those with successful extubation [19]. Thille et al. reported a comparable 32% mortality rate among high-risk patients experiencing extubation failure within the ICU stay [12]. In our cohort of 165 patient with cirrhosis who survived IMV and reached extubation, we observed in-hospital mortality rates of 47% and 7%, respectively, according to extubation failure or success (*p* < 0.01). There also was a trend for higher in-hospital mortality when comparing extubation failure patients with and without cirrhosis (47% versus 31%, *p* = 0.06). ICU and in-hospital lengths of stay were significantly longer in patients with cirrhosis who faced extubation failure in comparison to those without cirrhosis (Table S2). These findings are in accordance with the generally poor outcomes of patients with cirrhosis admitted to the ICU [2, 3, 5, 6, 21].

To our knowledge, we report for the first time causes of extubation failure at 48 h in selected patients with cirrhosis. The primary cause of reintubation was neurologic failure (36%), followed by non-cardiac respiratory failure (21%). In contrast, among patients without cirrhosis, the primary causes of reintubation at 48 h encompassed hemodynamic instability (30%), non-cardiac respiratory failure (24%), upper airway obstruction (10%), and neurologic failure (10%) (Table S4).

One of the secondary objectives of the study was to identify specific independent risk factors for extubation failure in patients with cirrhosis. We found that only admission for shock was associated with extubation failure in patients with cirrhosis **(**Table 4**)**. This aligns with the observations of Gibbs et al. who previously showed that cirrhotic patients intubated for respiratory failure or cardiac instability faced a significantly lower hazard ratio of successful extubation [8]. In contrast, we identified the same risk factors as previously reported in patients without cirrhosis: severity scores before extubation (SOFA ≥ 8), admission for acute respiratory failure or neurologic failure, and absence of strong cough [19, 29, 30].

The study has certain limitations and strengths requiring discussion. First, the classification of the primary causes of reintubation was a challenge. To address this challenge, a strategy to minimize biases was implemented: in each participating ICU, two individuals assessed the primary cause of extubation failure. Disagreement was adjudicated by two independent observers [22]. Second, this study reflected French ICU practices between 2013 and 2015 which may have evolved due to publication of national and international guidelines for weaning and extubation [10, 11]. Notably, HFNO was mostly unused during this period in the participating centers. Third, our study included consecutive patients under IMV both with and without cirrhosis. Whether the cause of admission was related to a cirrhosis decompensation was not documented. Some patients were likely admitted for other reasons and may have a history of stable cirrhosis. While our study did not differentiate between stable cirrhosis and ACLF, it is plausible that the presence of ACLF increases the risk of extubation failure due to factors such as encephalopathy and ascites. However, patients with cirrhosis admitted to ICU, whatever the cause, are at risk of severe decompensation and ACLF. The absence of detailed liver disease characterization also limits our ability to fully assess the impact of cirrhosis severity on extubation failure. Future studies should prioritize collecting components of Child–Pugh and MELD scores, and ACLF stratification to better assess extubation outcomes in this population. Finally, the FREE-REA study excluded patients with do-not-reintubate order, potentially underestimating the true reintubation rate, particularly in patients with cirrhosis where the risk–benefit assessment of intubation is often more complex. Therefore, while our findings are relevant to patients deemed eligible for reintubation, they may not fully reflect the spectrum of outcomes in all critically ill cirrhotic patients, where decisions regarding life-sustaining treatments are highly individualized.

Our research emphasizes the necessity for a robust agenda directed at further investigating the underlying mechanisms and risk factors specific to extubation failure in patients admitted in ICU for decompensated cirrhosis. Future prospective studies should specifically focus on exploring the efficacy of adapted prophylactic measures. Additionally, multicenter trials could provide broader insights into the best practices for managing such high-risk patients. Ultimately, enhancing our understanding of extubation challenges in patients with cirrhosis may lead to improved clinical guidelines and patient outcomes in the critical care setting.

## Conclusion

In this *post-hoc* analysis of a large multicenter cohort of 1,443 critically ill patients, the incidence of reintubation within 7 days after extubation was 1.6-fold higher in patients with cirrhosis compared to those without cirrhosis. In patients with cirrhosis, neurologic failure was the main cause for reintubation at 48 h. Extubation failure was associated with longer ICU and hospital lengths of stay, with a trend for higher in-hospital mortality in patients with cirrhosis. These findings require confirmation through dedicated prospective studies.

## Supplementary Information

Additional file 1.

## Data Availability

Research data and other material (e.g., study protocol and statistical analysis plan) will be made available to the scientific community, immediately on publication, with as few restrictions as possible. All requests should be submitted to the corresponding author who will review with the other investigators for consideration. A data use agreement will be required before the release of participant data and institutional review board approval as appropriate.

## Abbreviations

CI: Confidence interval
COPD: Chronic obstructive pulmonary disease
HFNO: High flow nasal oxygen
ICU: Intensive care unit
IRB: International review board
MV: Mechanical ventilation
NIV: Non-invasive ventilation
OR: Odds ratio
SBT: Spontaneous breathing trial
SOFA: Sequential organ failure assessment
