# Supplementary material for: Incidence and outcomes of extubation failure in mechanically ventilated patients with cirrhosis: a post-hoc analysis of a prospective multicenter study
Source: Ann Intensive Care. 2025 Oct 16;15:160. doi: 10.1186/s13613-025-01576-3 (PMC12532547; doi:10.1186/s13613-025-01576-3)
Supplement: Supplementary file 1 — Additional file 1. [file 13613_2025_1576_MOESM1_ESM.docx]

**ELECTRONIC SUPPLEMENTARY MATERIAL SUMMARY**

**Figure S1. Cumulative incidence of mortality within the 60 days following extubation – Sensitivty analysis**

1. Cumulative incidence of mortality within the 60 days following extubation among patients **with cirrhosis and length of mechanical ventilation ≤ 24 hours** compared between those who required reintubation (red) and those who were successfully extubated (blue)
2. Cumulative incidence of mortality within the 60 days following extubation among patients with **length of mechanical ventilation ≤ 24 hours** compared between patients with cirrhosis (red) and those without cirrhosis (blue) who required reintubation
3. Cumulative incidence of mortality within the 60 days following extubation among patients **with cirrhosis and length of mechanical ventilation > 24 hours** compared between those who required reintubation (red) and those who were successfully extubated (blue)
4. Cumulative incidence of mortality within the 60 days following extubation among patients with **length of mechanical ventilation > 24 hours** compared between patients with cirrhosis (red) and those without cirrhosis (blue) who required reintubation
5. Cumulative incidence of mortality within the 60 days following extubation among patients **with cirrhosis and post-operative reason for ICU admission** compared between those who required reintubation (red) and those who were successfully extubated (blue)
6. Cumulative incidence of mortality within the 60 days following extubation among patients with **post-operative reason for ICU admission** compared between patients with cirrhosis (red) and those without cirrhosis (blue) who required reintubation
7. Cumulative incidence of mortality within the 60 days following extubation among patients **with cirrhosis and medical reason for ICU admission** compared between those who required reintubation (red) and those who were successfully extubated (blue)
8. Cumulative incidence of mortality within the 60 days following extubation among patients with **medical reason for ICU admission** compared between patients with cirrhosis (red) and those without cirrhosis (blue) who required reintubation

**Table S1** **Baseline characteristics in 1443 consecutive patients with and without cirrhosis undergoing a first extubation procedure in ICU**

**Table S2 Secondary outcomes among extubation failure patients with and without cirrhosis**

**Table S3 Secondary outcomes in patients overall, with cirrhosis and without cirrhosis**

**Table S4. Causes of reintubation at 48 hours among patients overall, with and without cirrhosis**

**Table S5. Parameters during and after extubation and spontaneous breathing trial according to extubation failure**

**Table S6. Baseline characteristics in patients with cirrhosis who experienced extubation failure at day 2 and between day 3 and day 7**

**Table S7. Secondary outcomes in patients with cirrhosis who experienced extubation failure at day 2 and between day 3 and day 7**

**Figure S1. Cumulative incidence of mortality within the 60 days following extubation – Sensitivity analysis**

1. Cumulative incidence of mortality within the 60 days following extubation among patients **with cirrhosis and length of mechanical ventilation ≤ 24 hours** compared between those who required reintubation (red) and those who were successfully extubated (blue)


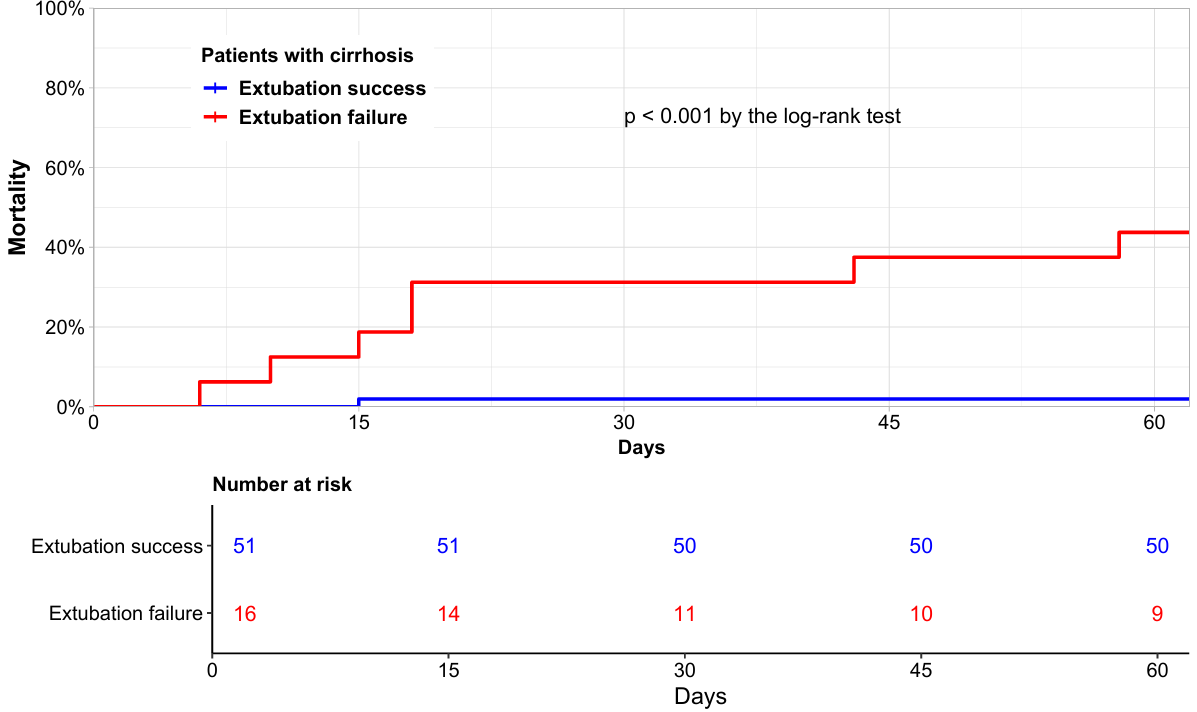


1. Cumulative incidence of mortality within the 60 days following extubation among patients with **length of mechanical ventilation ≤ 24 hours** compared between patients with cirrhosis (red) and those without cirrhosis (blue) who required reintubation


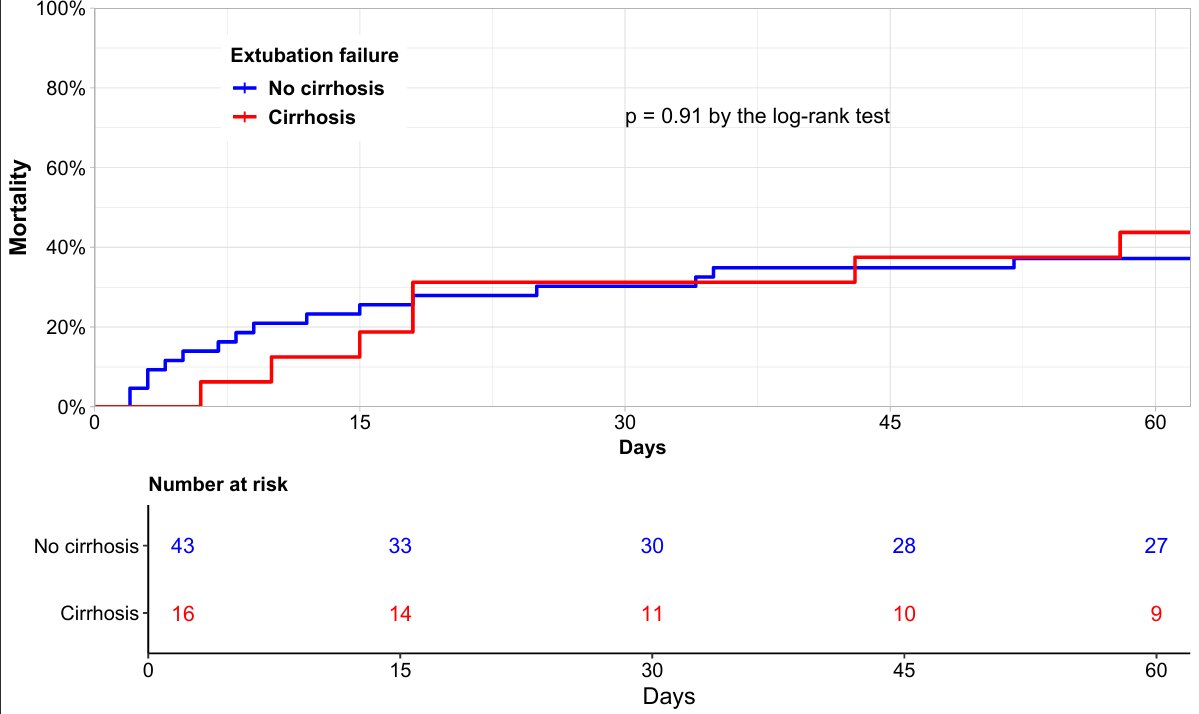


1. Cumulative incidence of mortality within the 60 days following extubation among patients **with cirrhosis and length of mechanical ventilation > 24 hours** compared between those who required reintubation (red) and those who were successfully extubated (blue)


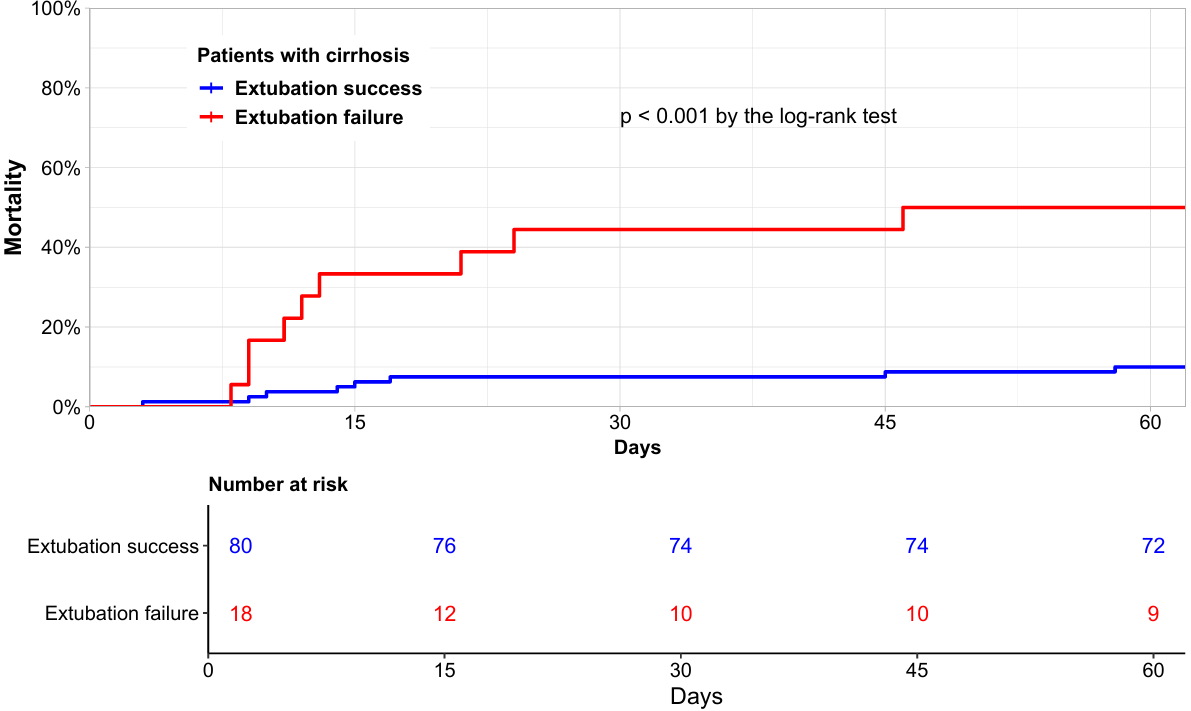


1. Cumulative incidence of mortality within the 60 days following extubation among patients with **length of mechanical ventilation > 24 hours** compared between patients with cirrhosis (red) and those without cirrhosis (blue) who required reintubation


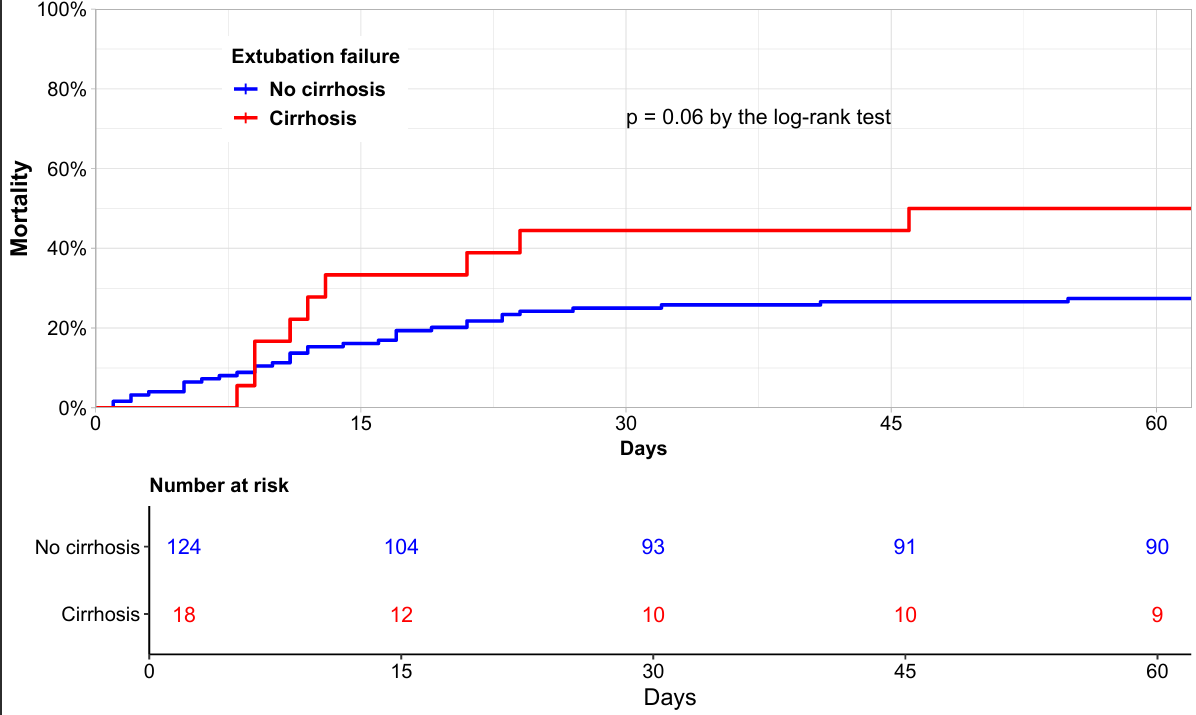


1. Cumulative incidence of mortality within the 60 days following extubation among patients **with cirrhosis and post-operative reason for ICU admission** compared between those who required reintubation (red) and those who were successfully extubated (blue)


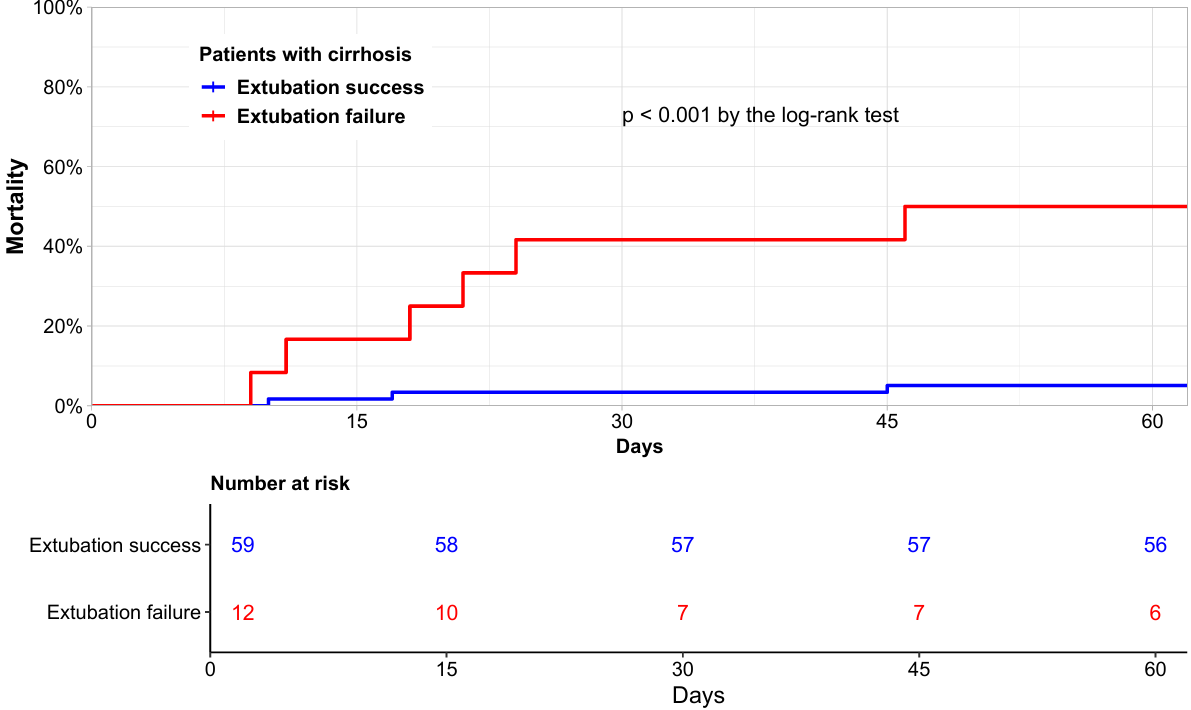


1. Cumulative incidence of mortality within the 60 days following extubation among patients with **post-operative reason for ICU admission** compared between patients with cirrhosis (red) and those without cirrhosis (blue) who required reintubation


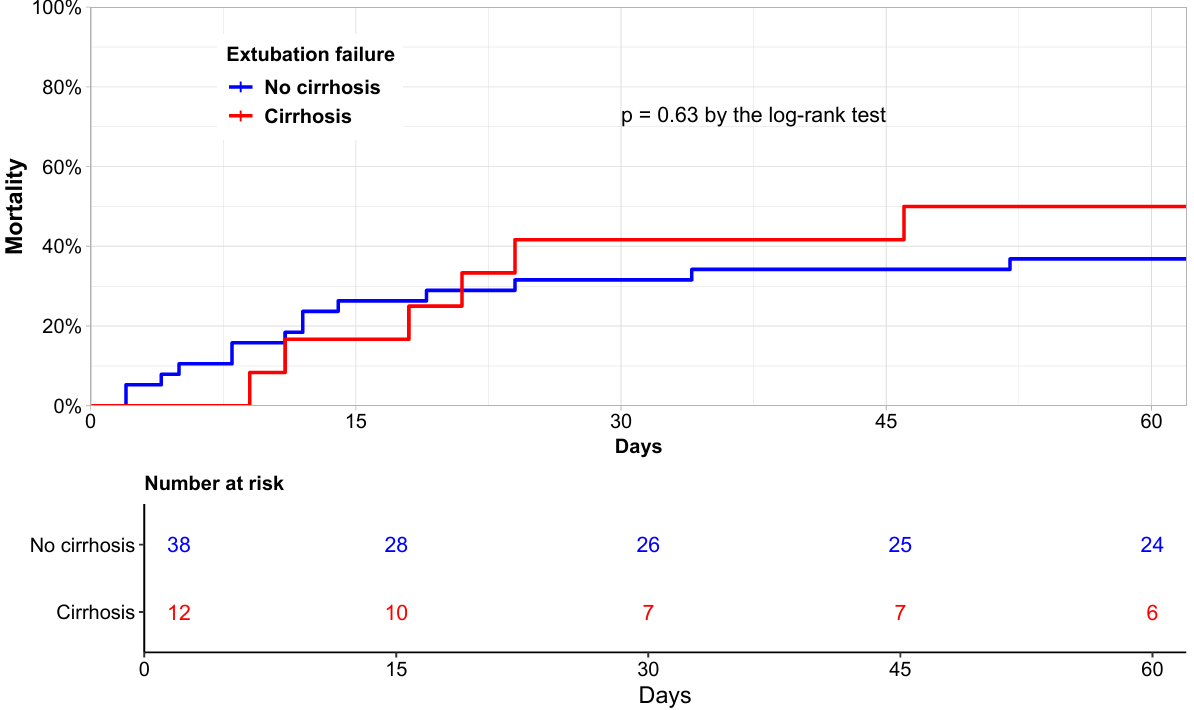


1. Cumulative incidence of mortality within the 60 days following extubation among patients **with cirrhosis and medical reason for ICU admission** compared between those who required reintubation (red) and those who were successfully extubated (blue)


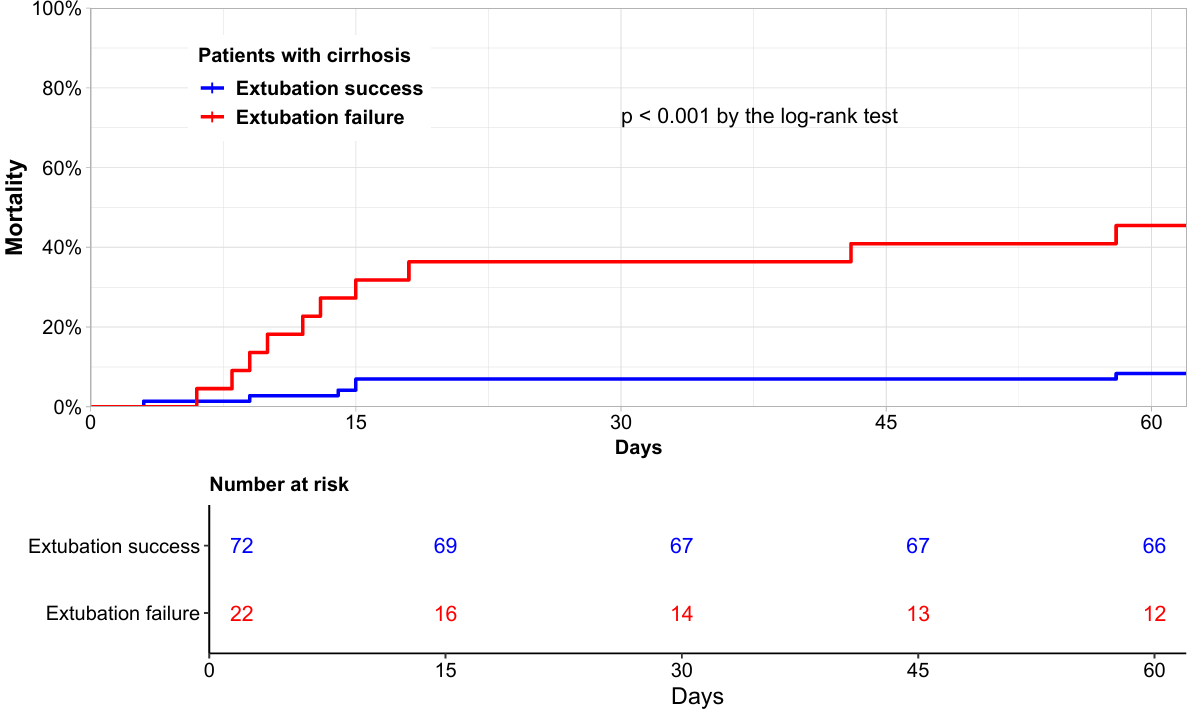


1. Cumulative incidence of mortality within the 60 days following extubation among patients with **medical reason for ICU admission** compared between patients with cirrhosis (red) and those without cirrhosis (blue) who required reintubation


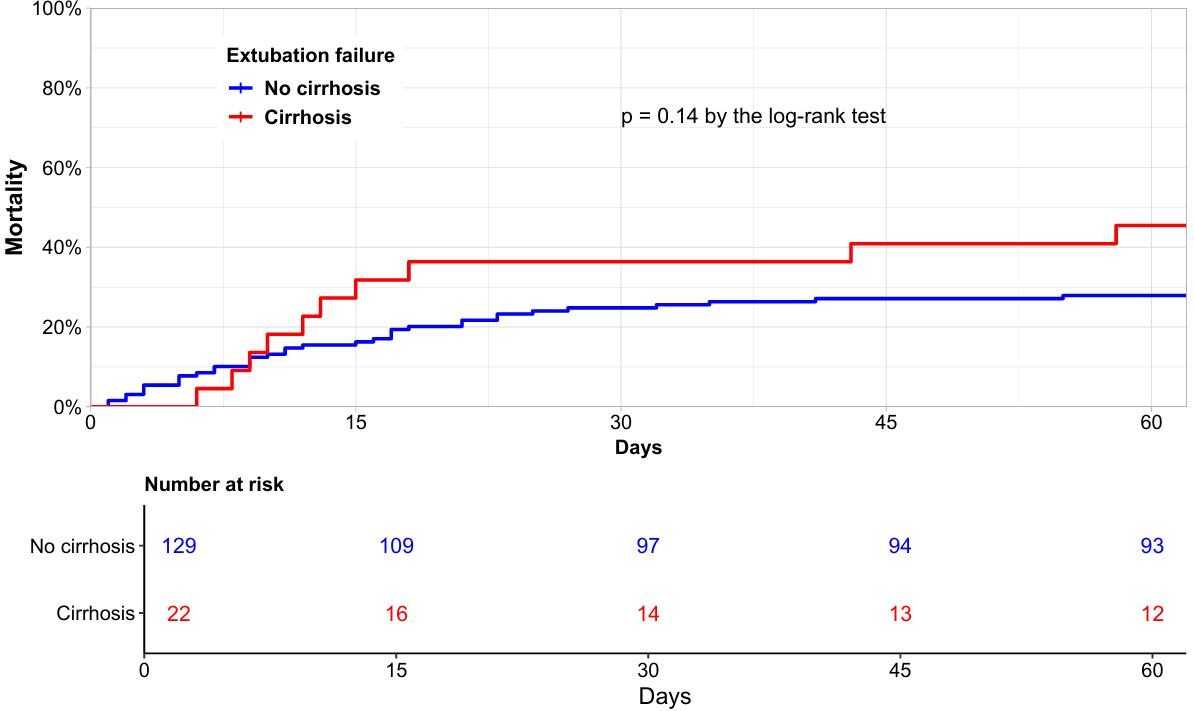


BMI Body Mass Index, COPD Chronic Obstructive Pulmonary Disease, IQR Interquartile range ICU Intensive Care Unit, SAPS2: Simplified Acute Physiology Score, SD Standard deviation, SOFA: Sequential Organ Failure Assessment

| **Table S1.** **Baseline characteristics in 1443 consecutive patients with and without cirrhosis undergoing a first extubation procedure in ICU** | | | | |  |
| --- | --- | --- | --- | --- | --- |
| **Characteristic** | **Study population (n=1443)** |  | | | |
|  |  | **Patients with cirrhosis (n=165)** | **Patients without cirrhosis (n=1278)** | **p-value** | |
| Age, mean (SD) | 59 (17) | 58 (10) | 59 (17) | 0.20 | |
| Female sex, n (%) | 531 (37%) | 43 (26%) | 488 (38%) | <0.001 | |
| SAPS2, mean (SD) | 45 (18) | 45 (15) | 45 (18) | 0.95 | |
| SOFA score before extubation, mean (SD) | 3 (4) | 6 (6) | 3 (3) | <0.001 | |
| BMI (kg/m²), mean (SD) | 26.4 (6.3) | 26.1 (4.8) | 26.4 (6.5) | 0.32 | |
| BMI ≥ 30kg/m², n (%) | 293 (20%) | 28 (17%) | 265 (21%) | 0.40 | |
| Type of admission |  |  |  | 0.57 | |
| Medical, n (%) | 799 (55%) | 87 (53%) | 712 (56%) |  | |
| Surgical, n (%) | 644 (45%) | 78 (47%) | 566 (44%) |  | |
| Smoking, n (%) | 365 (25%) | 59 (36%) | 306 (24%) | <0.001 | |
| Alcohol abuse, n (%) | 319 (25%) | 101 (61%) | 218 (17%) | <0.001 | |
| COPD, n (%) | 184 (13%) | 26 (16%) | 158 (12%) | 0.27 | |
| Chronic renal disease, n (%) | 173 (12%) | 38 (23%) | 135 (11%) | <0.001 | |
| Chronic heart disease, n (%) | 147 (10%) | 13 (7.9%) | 134 (10%) | 0.28 | |
| Primary reason for ICU admission, n (%) |  |  |  |  | |
| Acute respiratory failure | 318 (22%) | 25 (15%) | 293 (23%) | 0.03 | |
| Post-operative | 497 (34%) | 71 (43%) | 426 (33%) | 0.02 | |
| Neurologic failure | 323 (22%) | 20 (12%) | 303 (24%) | <0.001 | |
| Hemodynamic instability | 261 (18%) | 34 (21%) | 227 (18%) | 0.43 | |
| Others | 44 (4%) | 15 (9%) | 29 (2%) | <0.001 | |
| Primary reason for intubation, n (%) |  |  |  |  | |
| Acute respiratory failure | 332 (23%) | 35 (21%) | 297 (23%) | 0.63 | |
| Neurologic failure | 339 (23%) | 25 (15%) | 314 (25%) | <0.01 | |
| Hemodynamic instability | 157 (11%) | 16 (10%) | 141 (11%) | 0.70 | |
| Cardiac arrest | 51 (4%) | 2 (1%) | 49 (4%) | 0.14 | |
| Surgery | 475 (33%) | 57 (35%) | 418 (32%) | 0.67 | |
| Others | 89 (6%) | 30 (18%) | 59 (4%) | <0.001 | |
| Length of intubation before extubation (days), median (IQR) | 2 [1-5] | 2 [1-5] | 2 [1-6] | 0.11 | |

**Table S2. Secondary outcomes among extubation failure patients with and without cirrhosis**

| **Characteristic** | **Overall (n=201)** | **Extubation failure in patients with cirrhosis (n=34)** | **Extubation failure in**  **patients without cirrhosis**  **(n=167)** | **p-value** |
| --- | --- | --- | --- | --- |
| Total length of IMV (days), median (IQR) | 10 [4-16] | 10 [3-16] | 10 [3-15] | 0.84 |
| Hospital-acquired infections, n (%) | 88 (44%) | 22 (65%) | 66 (40%) | <0.01 |
| Pneumonia | 48 (24%) | 12 (35%) | 36 (22%) | 0.13 |
| Catheter | 5 (2%) | 0 (0%) | 5 (3%) | 0.68 |
| Bloodstream | 22 (11%) | 6 (18%) | 16 (10%) | 0.28 |
| Urinary tract | 13 (7%) | 4 (12%) | 9 (5%) | 0.32 |
| Use of vasopressor, n (%) | 58 (29%) | 17 (50%) | 41 (25%) | <0.01 |
| Use of renal replacement therapy, n (%) | 43 (22%) | 6 (18%) | 37 (22%) | 0.47 |
| Use of curative NIV after extubation, n (%) | 79 (40%) | 16 (47%) | 63 (38%) | 0.31 |
| ICU length of stay (days), median (IQR) | 16 [10-27] | 17 [12-36] | 16 [10-24] | 0.03 |
| In-hospital length of stay (days), median (IQR) | 27 [15-46] | 34 [18-58] | 25 [14-41] | <0.01 |
| In hospital mortality, n (%) | 67 (33%) | 16 (47%) | 51 (31%) | 0.06 |

ICU: Intensive Care Unit, IMV Invasive Mechanical Ventilation, IQR Interquartile range, NIV Non Invasive Ventilation

“Total length of IMV” refers to the cumulative duration of invasive mechanical ventilation during the entire ICU stay. “Use of vasopressor” and “Use of renal replacement therapy” refers to the initiation or continuation of these supports exclusively after extubation and until ICU discharge. “Hospital-acquired infections” were recorded if they occurred after extubation.

**Table S3 Secondary outcomes in patients overall, with cirrhosis and without cirrhosis**

| **Characteristic** | **Overall (n=1443)** | **Patients with cirrhosis**  **(n=165)** | **Patients without cirrhosis**  **(n=1278)** | **p-value** |
| --- | --- | --- | --- | --- |
| Total length of IMV (days), mean (SD) | 3 [1-8] | 3 [1-9] | 3 [1-8] | 0.43 |
| Hospital-acquired infections, n (%) | 253 (18%) | 45 (27%) | 208 (16%) | <0.01 |
| Pneumonia | 106 (7%) | 16 (10%) | 90 (7%) | <0.01 |
| Catheter | 28 (2%) | 4 (2%) | 24 (2%) | 0.86 |
| Bloodstream | 80 (6%) | 17 (10%) | 63 (5%) | <0.01 |
| Urinary tract | 39 (3%) | 8 (5%) | 31 (2%) | 0.07 |
| Use of vasopressor, n (%) | 135 (9%) | 29 (18%) | 106 (8%) | <0.01 |
| Use of renal replacement therapy, n (%) | 68 (5%) | 12 (7%) | 56 (4%) | 0.09 |
| Use of curative NIV after extubation, n (%) | 424 (29%) | 49 (30%) | 375 (29%) | 0.93 |
| ICU length of stay (days), median (IQR) | 7 [3-15] | 10 [5-16] | 7 [3-15] | <0.01 |
| In-hospital length of stay (days), median (IQR)) | 19 [9-32] | 25 [17-46] | 17 [9-31] | <0.01 |
| In-hospital mortality, n (%) | 170 (12%) | 25 (15%) | 145 (11%) | 0.15 |

ICU: Intensive Care Unit, IMV Invasive Mechanical Ventilation, IQR Interquartile range, NIV Non Invasive Ventilation,

“Total length of IMV” refers to the cumulative duration of invasive mechanical ventilation during the entire ICU stay. “Use of vasopressor” and “Use of renal replacement therapy” refers to the initiation or continuation of these supports exclusively after extubation and until ICU discharge. “Hospital-acquired infections” were recorded if they occurred after extubation.

**Table S4. Causes of** **reintubation at 48 hours among patients overall, with and without cirrhosis**

| **Characteristic** | **Overall (n=146)** | **Patients with cirrhosis (n=14)** | **Patients without cirrhosis (n=132)** | **p-value** |
| --- | --- | --- | --- | --- |
| Primary cause of reintubation^*^ |  |  |  |  |
| Respiratory failure, n (%) | 35/146 (24%) | 3/14 (21%) | 32/132 (24%) | 0.81 |
| Upper airway obstruction, n (%) | 13/146 (9%) | 0/14 (0%) | 13/132 (10%) | 0.23 |
| Acute pulmonary edema, n (%) | 7/146 (5%) | 0/14 (0%) | 7/132 (5%) | 0.38 |
| Hemodynamic instability, n (%) | 41/146 (29%) | 2/14 (14%) | 39/132 (30%) | 0.23 |
| Cardiac arrest, n (%) | 5/146 (3%) | 0/14 (0%) | 5/132 (4%) | 0.46 |
| Neurologic failure, n (%) | 18/146 (12%) | 5/14 (36%) | 13/132 (10%) | 0.01 |
| Other, n (%) | 27/146 (18%) | 4/14 (29%) | 23/132 (17%) | 0.31 |

^*^Causes are reported for patients who experienced reintubation at 48 hours (n=146)

**Table S5. Parameters during and after extubation and spontaneous breathing trial according to extubation failure**

| **Characteristic** | **Overall (n=1443)** | **Patients with cirrhosis (n=165)** | | | | **Patients without cirrhosis (n=1278)** | | | | **Overall**  **p-value** |
| --- | --- | --- | --- | --- | --- | --- | --- | --- | --- | --- |
|  |  | **Overall**  **(165)** | **Extubation success (n=131)** | **Extubation failure (n=34)** | **p-value** | **Overall**  **(1278)** | **Extubation success (n=1111)** | **Extubation failure (n=167)** | **p-value** |  |
| **Evaluation before extubation** |  |  |  |  |  |  |  |  |  |  |
| Agitation | 269 (19%) | 26 (16%) | 18 (14%) | 8 (24%) | 0.26 | 243 (19%) | 213 (19%) | 30 (18%) | 0.79 | 0.37 |
| Response to simple orders | 1258 (87%) | 146 (88%) | 114 (87%) | 32 (94%) | 0.39 | 1112 (87%) | 968 (87%) | 144 (86%) | 0.84 | 0.68 |
| Eye-tracking | 1145 (79%) | 134 (81%) | 106 (81%) | 28 (82%) | 1.00 | 1011 (79%) | 883 (79%) | 128 (77%) | 0.46 | 0.60 |
| Moderate to strong cough | 1077 (75%) | 133 (81%) | 108 (82%) | 25 (74%) | 0.35 | 944 (74%) | 831 (75%) | 113 (68%) | 0.06 | 0.08 |
| Corticosteroids before extubation | 128 (9%) | 17 (10% | 15 (11%) | 2 (5.9%) | 0.53 | 111 (9%) | 93 (8.4%) | 18 (11%) | 0.38 | 0.59 |
| **Spontaneous breathing trial** | 1145 (79%) | 124 (75%) | 99 (75%) | 25 (74%) | 0.81 | 1021 (80%) | 884 (80%) | 137 (82%) | 0.46 | 0.22 |
| **Without PS** | 535 (37%) | 39 (24%) | 34 (26%) | 5 (15%) | 0.17 | 496 (39%) | 416 (37%) | 80 (48%) | 0.01 | <0.01 |
| T-tube | 505 (35%) | 32 (19%) | 27 (21%) | 5 (15%) | 0.52 | 473 (37%) | 397 (36%) | 76 (46%) | 0.02 | <0.01 |
| PEEP 0 – PS 0 | 11 (1%) | 3 (2%) | 3 (2%) | 0 (0%) | 0.61 | 8 (1%) | 7 (1%) | 1 (1%) | 0.96 | 0.16 |
| PEEP only | 19 (1%) | 4 (2%) | 4 (2%) | 0 (0%) | 0.58 | 15 (1%) | 12 (1.1%) | 3 (1.8%) | 0.51 | 0.29 |
| **With PS** | 610 (42%) | 85 (51%) | 65 (49%) | 20 (59%) | 0.34 | 525 (41%) | 468 (42%) | 57 (34%) | 0.06 | <0.01 |
| PS only | 199 (14%) | 40 (24%) | 33 (25%) | 7 (21%) | 0.58 | 159 (12%) | 141 (13%) | 18 (11%) | 0.53 | <0.01 |
| PEEP and PS | 411 (28%) | 45 (27%) | 32 (24%) | 13 (38%) | 0.11 | 366 (29%) | 327 (29%) | 39 (23%) | 0.11 | 0.71 |
| **Precautions for reintubation** |  |  |  |  |  |  |  |  |  |  |
| FiO2 set at 100% before  extubation | 438 (30%) | 43 (26%) | 37 (28%) | 6 (18%) | 0.21 | 395 (31%) | 343 (31%) | 52 (31%) | 1.00 | 0.48 |
| Reintubation kit ready | 536 (37%) | 28 (17%) | 25 (19%) | 3 (8.8%) | 0.24 | 508 (40%) | 443 (40%) | 65 (39%) | 0.88 | <0.01 |
| Physiotherapy after extubation | 726 (50%) | 72 (44%) | 57 (44%) | 15 (44%) | 1.00 | 654 (51%) | 546 (49%) | 108 (65%) | <0.01 | 0.08 |
| Prophylactic NIV in the hour after extubation | 329 (23%) | 23 (14%) | 17 (13%) | 6 (18%) | 0.48 | 306 (24%) | 252 (23%) | 54 (32%) | <0.01 | <0.01 |
| **Adverse events in the hour after extubation** | 403 (28%) | 26 (16%) | 15 (11%) | 11 (32%) | <0.01 | 377 (29%) | 281 (25%) | 96 (57%) | <0.01 | <0.01 |
| Hypoxemia | 95 (7%) | 9 (6%) | 7 (5%) | 2 (6%) | 1.00 | 86 (7%) | 52 (5%) | 34 (20%) | <0.01 | 0.65 |
| Tachypnea | 167 (12%) | 10 (6%) | 6 (5%) | 4 (12%) | 0.24 | 157 (12%) | 108 (10%) | 49 (29%) | <0.01 | 0.03 |
| Lower airway obstruction | 183 (12%) | 10 (6%) | 6 (5%) | 4 (12%) | 0.24 | 173 (14%) | 121 (11%) | 52 (31%) | <0.01 | 0.01 |
| Upper airway obstruction | 33 (2%) | 1 (1%) | 0 (0%) | 1 (3%) | 0.47 | 32 (2%) | 15 (1%) | 17 (10%) | <0.01 | 0.20 |
| Collapsus (SAP < 80mmHg) | 30 (2%) | 2 (1%) | 1 (1%) | 1 (3%) | 0.88 | 28 (2%) | 17 (2%) | 11 (7%) | <0.01 | 0.59 |
| Acute pulmonary edema | 12 (1%) | 0 (0%) | 0 (0%) | 0 (0%) | 1.00 | 12 (1%) | 8 (1%) | 4 (2%) | 0.01 | 0.43 |
| Tachycardia | 188 (13%) | 10 (6%) | 6 (5%) | 4 (12%) | 0.24 | 178 (14%) | 136 (12%) | 42 (25%) | <0.01 | <0.01 |
| Cardiac arrest | 6 (<1%) | 0 (0%) | 0 (0%) | 0 (0%) | 1.00 | 6 (<1%) | 3 (0.3%) | 3 (1.8%) | 0.04 | 0.81 |
| Agitation | 18 (1%) | 1 (1%) | 0 (0%) | 1 (3%) | 0.21 | 17 (1%) | 0 (0%) | 17 (10%) | <0.01 | 0.64 |
| Neurologic failure | 21 (1%) | 0 (0%) | 0 (0%) | 0 (0%) | 1.00 | 21 (2%) | 6 (0.5%) | 15 (9%) | <0.01 | 0.19 |

PS: Pressure support, PEEP: Positive End-Expiratory Pressure, NIV: Noninvasive Ventilation, SAP: Systolic Arterial Pressure

| **Table S6. Baseline characteristics in patients with cirrhosis who experienced extubation failure at day 2 and between day 3 and day 7** | | | | |  |
| --- | --- | --- | --- | --- | --- |
| **Characteristic** | **Overall**  **(n=34)** |  | | | |
|  |  | **Extubation failure at day 2**  **(n=14)** | **Extubation failure between day 3 and day 7**  **(n=20)** | **p-value** | |
| Age, mean (SD) | 59 (12) | 58 (11) | 60 (12) | 0.62 | |
| Female sex, n (%) | 10 (29%) | 2 (14%) | 8 (40%) | 0.22 | |
| SAPS2, mean (SD) | 48 (13) | 43 (10) | 52 (15) | 0.07 | |
| SOFA score before extubation, mean (SD) | 6 (5) | 6 (5) | 6 (5) | 0.75 | |
| BMI (kg/m²), mean (SD) | 26.0 (5.1) | 25.3 (5.3) | 27.0 (4.0) | 0.34 | |
| BMI ≥ 30kg/m², n (%) | 6 (18%) | 2 (14%) | 4 (20%) | 0.89 | |
| Type of admission |  |  |  | 0.82 | |
| Medical, n (%) | 19 (56%) | 7 (50%) | 12 (60%) |  | |
| Surgical, n (%) | 15 (44%) | 7 (50%) | 8 (40%) |  | |
| Smoking, n (%) | 12 (35%) | 6 (43%) | 6 (30%) | 0.68 | |
| Alcohol abuse, n (%) | 21 (62%) | 11 (79%) | 10 (50%) | 0.18 | |
| COPD, n (%) | 3 (8.8%) | 1 (7.1%) | 2 (10%) | 1.00 | |
| Chronic renal disease, n (%) | 11 (32%) | 4 (29%) | 7 (35%) | 0.98 | |
| Chronic heart disease, n (%) | 1 (2.9%) | 0 (0%) | 1 (5.0%) | 1.00 | |
| Primary reason for ICU admission, n (%) |  |  |  |  | |
| Acute respiratory failure | 1 (2.9%) | 1 (7.1%) | 0 (0%) | 0.86 | |
| Post-operative | 12 (35%) | 3 (21%) | 9 (45%) | 0.29 | |
| Neurologic failure | 3 (8.8%) | 2 (14%) | 1 (5.0%) | 0.74 | |
| Hemodynamic instability | 13 (38%) | 6 (43%) | 7 (35%) | 0.92 | |
| Others | 5 (15%) | 2 (14%) | 3 (15%) | 0.87 | |
| Primary reason for intubation, n (%) |  |  |  |  | |
| Acute respiratory failure | 9 (26%) | 4 (29%) | 5 (25%) | 1.00 | |
| Neurologic failure | 6 (18%) | 3 (21%) | 3 (15%) | 0.98 | |
| Hemodynamic instability | 6 (18%) | 3 (21%) | 3 (15%) | 0.98 | |
| Cardiac arrest | 0 (0%) | 0 (0%) | 0 (0%) | 1.00 | |
| Surgery | 10 (29%) | 3 (21%) | 7 (35%) | 0.51 | |
| Others | 3 (9%) | 1 (7.1%) | 2 (10%) | 0.83 | |
| Length of intubation before extubation (days), mean (SD) | 2 [1-5] | 3 [1-6] | 1 [1-5] | 0.40 | |

BMI Body Mass Index, COPD Chronic Obstructive Pulmonary Disease, IQR Interquartile range ICU Intensive Care Unit, SAPS2: Simplified Acute Physiology Score, SD Standard deviation, SOFA: Sequential Organ Failure Assessment

**Table S7. Secondary outcomes in patients with cirrhosis who experienced extubation failure at day 2 and between day 3 and day 7**

| **Outcomes** | **Overall (n=34)** | **Extubation failure at day 2**  **(n=14)** | **Extubation failure between day 3 and day 7**  **(n=20)** | **p-value** |
| --- | --- | --- | --- | --- |
| Total length of IMV (days), median (IQR) | 10 (3-16) | 13 (3-18) | 10 (3-13) | 0.18 |
| Hospital-acquired infections, n (%) | 22 (65%) | 12 (86%) | 10 (50%) | 0.03 |
| Pneumonia | 12 (35%) | 6 (43%) | 6 (30%) | 0.68 |
| Catheter | 0 (0%) | 0 (0%) | 0 (0%) | 1.00 |
| Bloodstream | 6 (18%) | 3 (21%) | 3 (15%) | 0.98 |
| Urinary tract | 4 (12%) | 3 (21%) | 1 (5.0%) | 0.36 |
| Use of vasopressor, n (%) | 17 (50%) | 3 (21%) | 14 (70%) | 0.02 |
| Use of renal replacement therapy, n (%) | 6 (18%) | 1 (7.1%) | 5 (25%) | 0.38 |
| Use of curative NIV after extubation, n (%) | 16 (47%) | 5 (36%) | 11 (55%) | 0.45 |
| ICU length of stay (days), median (IQR) | 17 (12-36) | 16 (9-48) | 19 (14-32) | 0.95 |
| In-hospital length of stay (days), median (IQR) | 34 (16-58) | 42 (31-54) | 19 (15-62) | 0.52 |
| In hospital mortality, n (%) | 16 (47%) | 4 (29%) | 12 (60%) | 0.15 |

ICU: Intensive Care Unit, IMV Invasive Mechanical Ventilation, IQR Interquartile range, NIV Non Invasive Ventilation

“Total length of IMV” refers to the cumulative duration of invasive mechanical ventilation during the entire ICU stay. “Use of vasopressor” and “Use of renal replacement therapy” refers to the initiation or continuation of these supports exclusively after extubation and until ICU discharge. “Hospital-acquired infections” were recorded if they occurred after extubation.
